# Is Physical Performance a Differentiating Element between More or Less Successful Football Teams?

**DOI:** 10.3390/sports7100216

**Published:** 2019-09-30

**Authors:** Jose Antonio Asian Clemente, Bernardo Requena, Igor Jukic, Jack Nayler, Alfredo Santalla Hernández, Christopher Carling

**Affiliations:** 1 Football Science Institute, 18100 Granada, Spain; brequena@fsi-universitas.com (B.R.); igor.jukic@kif.hr (I.J.); jacknayler@hotmail.co.uk (J.N.); asanher@upo.es (A.S.H.); 2 Department of Sport sciences, Universidad Pablo de Olavide, 41704 Sevilla, Spain; 3 Department of Kinesiology of Sports, University of Zagreb, 10000 Zagreb, Croatia; 4 Celtic Football Club, Glasgow G40 3RE, UK; 5 Institute of Coaching and Performance, University of Central Lancashire, Greenbank Building, Preston PR1 2HE, UK; christopher.carling@gmail.com

**Keywords:** time-motion, match analysis, training, monitoring, demands

## Abstract

This study investigated the time-motion characteristics of football teams in the Spanish first division, in relation to their final competitive level as defined by league position (Champions League, Europa League, Upper mid-table, lower mid-table and relegation). Match observations (*n* = 9641) were collected using a multiple-camera computerized tracking system during the 2013–2014 competitive season. The following match parameters were analyzed: total distance, relative distance (m·min^−1^), distance < 14 km·h^−1^, >14 km·h^−1^, between 14–21 km·h^−1^, >21 km·h^−1^, and >24 km·h^−1^. Total distance and distance at different velocities (>14, 21, and 24 km·h^−1^) in and out of ball possession were also analyzed. A repeated analysis of variance and a comparison of effect sizes were carried out to compare the performance of the teams. The analysis of the data showed differences in physical performance characteristics between competitive levels. The volume of distance covered in the variables analyzed did not relate to success in soccer. Both successful and unsuccessful teams presented the same running requirements at higher velocities. These findings provide valuable information about the physical demands of the running requirements according to their final position in the league table.

## 1. Introduction

Over the past two decades, there has been a substantial increase in the knowledge of the running demands of professional soccer match play, through the use of time-motion analysis [1,2,3,4,5,6]. This can be associated with increased interest in this topic from coaching staff and the rapid development of computerized time-motion analysis systems. Indeed, contemporary time-motion analysis enables collection of valid, impartial, and objective information to aid monitoring and evaluation of the running performance of soccer players [7,8,9]. Traditionally, the amount of total distance covered, distance covered per minute or relative distance (m·min^−1^), distance covered in different speed zones, and the amount of accelerations and decelerations, have been used to assess the physical performance of soccer teams [2,3,9,10,11,12,13,14,15]. The scientific literature suggests that there are different physical requirements between teams, depending on various factors. For example, when a team plays against better-quality opponents, its players cover a greater total distance and distance covered above 14.4 km·h^−1^ in comparison to matches against lesser-quality opponents [15]. Similarly, different physical demands have been demonstrated when compared to competitive performance levels. In a study that analyzed the two best English leagues, it was found that teams in the English Football League Championship (2nd division) covered a greater total distance, distance covered above 19.8 km·h^−1^, and distance above 25.2 km·h^−1^, than their highest-level counterparts in the English Premier League (1st division) [12]. The positional role undertaken by a player also has a great impact on their movement demands. It has been shown that central defenders have the lowest running demands, whilst midfielders (central and wide) are the positions that cover the most total distance, whilst wide-midfielders and full-backs cover greater amounts of distance at higher velocities [3,12,14]. 

Although it may be thought that running demands are influenced by the team’s final league position, there is little scientific literature in regard to physical variables that differentiate successful and unsuccessful teams [3]. To date, only two studies have described the relationship between physical performance and a team’s final position in the standings of European competitive national leagues [12,13]. Using data collected from 2003–2004 to 2005–2006 in the Premier League, Di Salvo [12] found that players from less successful teams covered a greater global distance above 19.8 km·h^−1^ and 25.2 km·h^−1^, as well as a greater distance above 19.8 km·h^−1^ without possession of the ball, than players from the highest-ranked teams. In contrast, the authors did not observe any relationship between league position, the standing distance, and the distance above 19.8 km·h^−1^ with possession of the ball. In the second study, conducted in the Italian Serie A League (1st division), Rampinini et al. [13] compared the physical performance of the first five and last five teams in the 2003–2004 season. Players in the lower-ranked teams accumulated a greater total distance and distance covered above 14 km·h^−1^ and 19 km·h^−1^, whilst the higher-ranked teams accumulated a greater distance covered in all variables related to possession of the ball: total distance covered, distance covered above 14 km·h^−1^ and 19 km·h^−1^. Although these studies were carried out in two of the most important European leagues, some limitations should be considered. Firstly, both studies only considered two or three performance strata. This could be considered very general in the current understanding of elite football competitions, where one position up or down the table could determine success. Secondly, the competitive level of the leagues during those seasons could be questioned, as the final points difference between the 1st and the 3rd teams in the league were 14.0 ± 5.0 and 14.5 respectively. Additionally, in only one of the four seasons studied, did teams perform to a high standard (champion, finalist, or semi-finalist) in the competitions of the Union of European Football Associations (UEFA): Champions League and Europe League. 

In recent years, Spanish football has dominated European and World soccer. The national team has won one FIFA World Cup and two UEFA Euro championships, and club sides from Spain have won more UEFA Champions Leagues, UEFA Europa Leagues, and FIFA Club World Cups [9] than teams from any other country. Despite this, we have no evidence of any study that analyzes the time-motion demands of the Spanish league, where the teams’ high levels of international and domestic performance are precisely categorized. For these reasons, the aims of this study were to analyze the time-motion characteristics of teams in a highly competitive European national league and to compare them according to their competitive level as defined by final league position.

## 2. Materials and Methods

### 2.1. Experimental Approach to the Problem

**Figure 1:**
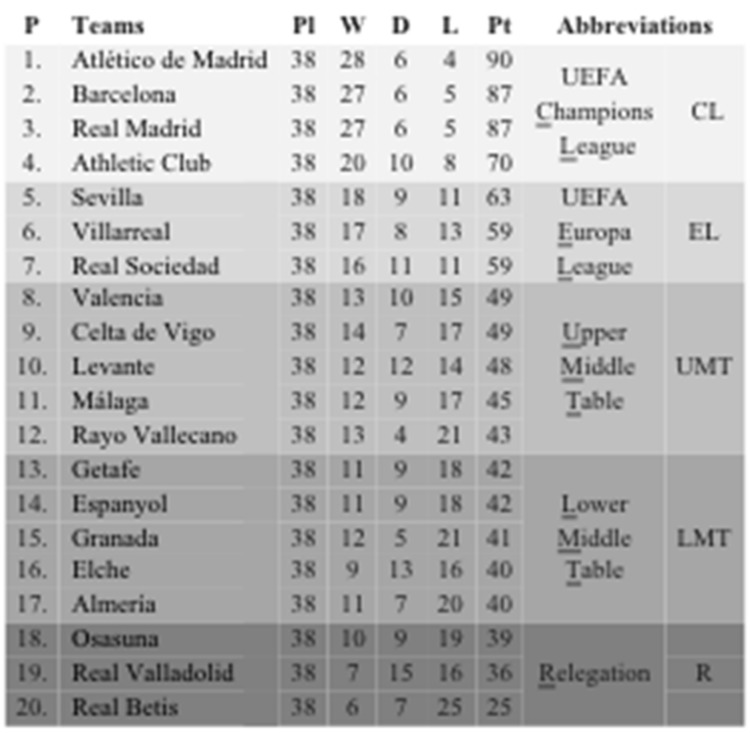
Organization of team groups according to the final classification in the season 2013–2014. P = position; Pl = played matches; W = matches won; D = matches Drawn; L = matches lost; Pt = points. Underlined letters represent the letters used to create the abbreviations of each group.

The current study was designed to examine the match play running performance of all the teams in the Spanish first division (La Liga) using a semi-automatic computerized player tracking system. Teams were placed into one of five categories based on their final league position of the 2013–2014 season (Figure 1). From the Champions league group, three out of the four teams were either champions, finalists, or quarterfinalists in the Champions League during the period studied. Similarly, in the Europa League, one of the team of this group was the champion, one of the Upper middle table teams was a quarterfinalist, and one of the relegated team reached the round of sixteen. This information indicates the strength of competition during the 2013–2014 season.

### 2.2. Sample

A total of 9641 individual data points from outfield players (excluding goalkeepers) were analyzed, with a median of 19.7 games per player (range = 1–38). The protocol for inclusion was previously described in literature [4]: (1) matches in which 90 min of play was completed; and (2) matches in which players played in their customary position throughout play, and the team’s playing formation remained unchanged. The experiment protocol was approved by the local Institutional Ethics Committee of the University of Pablo de Olavide, and was conducted according to the principles expressed in the Declaration of Helsinki.

### 2.3. Procedures

**Figure 2:**
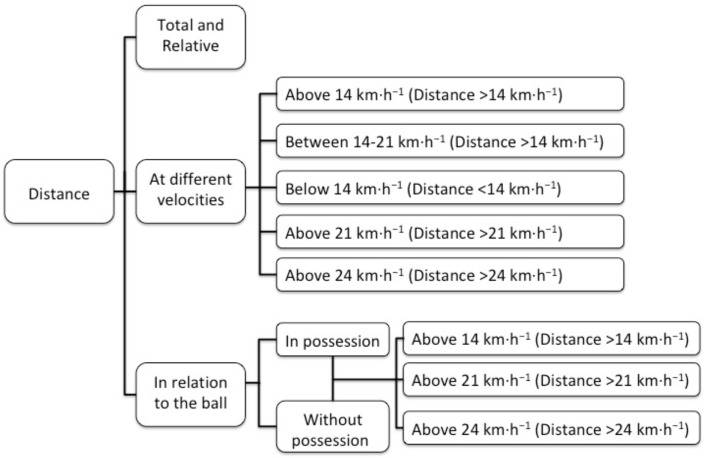
Variables analyzed to assess the locomotors demands of the matches.

A multi-camera, semi-automatic computerized player tracking system (MediaPro, Barcelona, Spain) was used to record the locomotors demands (velocities and distances) of match play (https://portal.mediacoach.es). Sixteen cameras placed high in the stadiums recorded the running performance of players, the system was in use by all teams of the 1st and 2nd division of the Spanish league [9]. Data collected were sent to a virtual server where coaches analyzed them. The use of this tracking system has appeared in previous research [9,16]. Utilizing trigonometry, the cameras captured the location of the players continuously, and the coaches downloaded the report post-hoc. The analyzed variables are displayed in Figure 2 and previously have been utilized in literature [10].

### 2.4. Statistical Analysis 

Data are presented as means ± standard deviation (SD). All variables presented a normal distribution (Shapiro–Wilk Test). A repeated-measures analysis of variance (ANOVA) was used to determine differences in each speed zone, in the distance covered, accelerations, decelerations, maximal velocity, and relative distance. Cohen’s effect size (ES) was also calculated in order to compare the magnitude of the differences between groups for certain variables [11]. Quantitative differences were assessed qualitatively [17] as: <1%, almost certainly not; 1−5%, very unlikely; 5−25%, unlikely; 25−75%, possible; 75−95%, probably; 95−99%, very likely; and >99%, almost certain. A substantial effect was set at >75% [14]. If the chance of higher or lower differences was >75%, data greater than this percentage were considered as a substantial effect between groups. The SPSS statistical software package (V20.0 for Windows, SPSS Inc., Chicago, IL, USA) was used for data analysis.

## 3. Results

**Figure 3:**
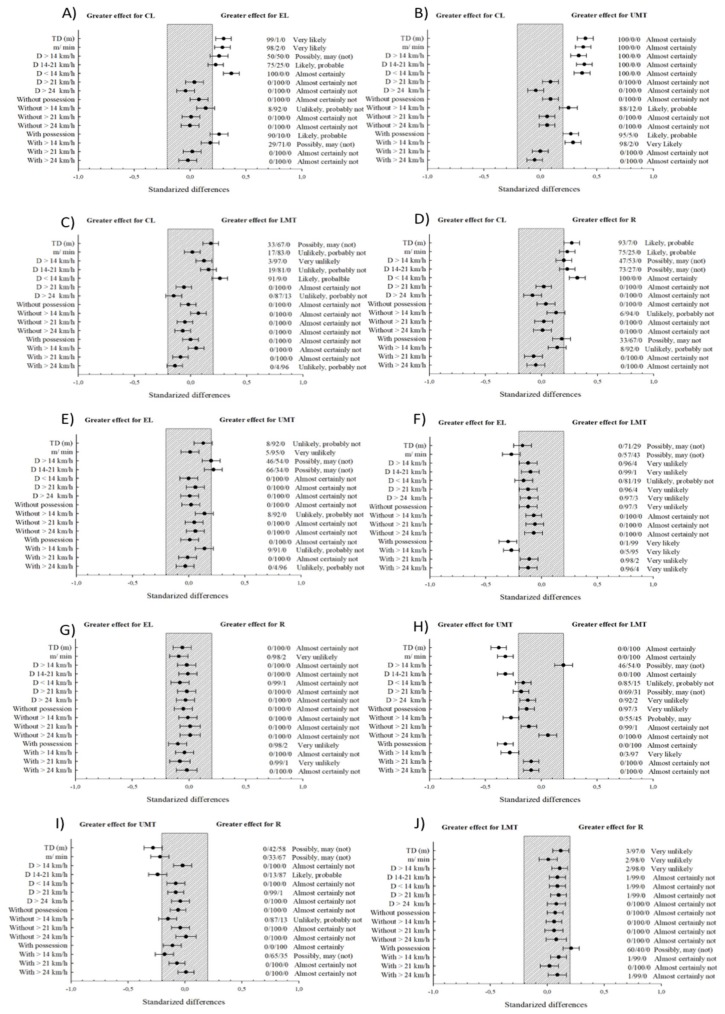
Comparison of the physical demands between groups. UEFA Champions League (CL), UEFA Europa League (EL), Upper Middle Table (UMT), Lower Middle Table (LMT) and Relegation (R). Bars indicate uncertainty in the true mean changes with 90% confidence intervals. The trivial area was calculated from the smallest worthwhile change (SWC).

The physical demands of the different groups of teams studied are shown in Table 1 and Figure 3. Similarly, the effect size and p values are shown in the Table 2.

In terms of total distance covered and relative distance, UMT, EL, and R teams accumulated the greatest values and showed substantial differences to CL teams. For distances >14 km·h^−1^ and between 14–21 km·h^−1^, the teams finishing between 8th and 12th in the league table obtained the greatest values, whilst teams between 1st and 4th positions accumulated the lowest amount of distance below 14 km·h^−1^ compared to all other teams. In the two highest velocity zones (distance >21 and >24 km·h^−1^), there were no differences between any of the groups of teams studied. Likewise, there were no differences between any of the teams’ values without the ball except for the distance covered without possession >14 km·h^−1^, where UMT teams showed a statistically greater amount of distance covered than the CL and LMT teams. Data for distance covered in possession of the ball showed that teams from the EL and UMT groups accumulated greater total distance and distance >14 km·h^−1^; whereas in the highest speed zone in possession, the top four teams in the league had the highest values. 

Table 2 shows the effect size comparison between groups. Data from this figure show small differences between groups, with trivial changes in most of the studied variables in relation to the smallest worthwhile change.

## 4. Discussion

The aim of this study was to analyze teams in a highly competitive European national league according to their competition level. The main findings were that: 

(1) Teams in the Spanish first division did not have large differences in match running demands when analyzed according to competitive level. (2) The amount of distance covered in the analyzed variables did not seem to relate to success in the population and time frame studied, as the teams that qualified for the highest level of competition, the Champions League (positions 1st–4th), did not obtain the highest values. (3) Successful and unsuccessful teams had the same running requirements at higher velocities (>21 and 24 km·h^−1^). 

Linking the success (or failure) of a team to physical performance has been of importance to coaches and strength and conditioning professionals. The analysis of the present data has demonstrated that the final position in the classification table of the Spanish La Liga did not depend on the running performance of the teams. These findings support the idea that overall, technical, and tactical effectiveness probably has a greater impact on results and a team’s final league ranking in soccer [3].

Although our general results are in line with those described in the Premier League [12] and Serie A [13], which shows different physical performances in teams at different competitive levels, our data shows some notable differences. Previous literature describes less successful teams as those that cover greater total distance and distance above 14 and 19 km·h^−1^ in Serie A [13], and greater distance above 19.8 and 25.2 km·h^−1^ in the English premier League [12]. Our results show that in La Liga during a competitive season, the worst teams (R and LMT) did not cover a greater distance than those teams who finished higher in the standings (CL, EL and UMT) in practically any of the variables studied. These findings contrast with Rampinini et al. [13] who showed that in Serie A, less successful teams (15th–20th in the final ranking) covered 4% greater total distance than the more successful teams (1st–5th in the final ranking). Our data showed no differences between the last three teams and the seven best teams in the final league table. In light of this, it seems that covering greater distances than other teams and maintaining higher mean speed during the matches is not sufficient to achieve a position amongst the top four teams in the league. 

The idea that a greater physical performance (running more meters) allows a team to win more matches should be discarded based on our data, as the most successful teams (CL) did not have the highest values for any of the previously mentioned variables or distances above and below 14 km·h^−1^, between 14–21 km·h^−1^, and above 21 and 24 km·h^−1^. This finding is backed up by the lack of difference between the second most successful group of teams (EL) and the teams at the bottom of the rankings who were relegated to the second Spanish division. With this in mind, we might suppose that the best teams in the league utilize technical and tactical means to win matches, and when a team is lower in the league it is probably not due to poor physical performance. 

Despite the fact that previous research in soccer has shown that the distance covered at higher velocities (high intensity running and sprinting) is an important indicator of performance [9,10,15] and influences league position [12,13], the present research did not replicate these findings. These differences are possibly caused by the more precise classifications of performance groupings (CL, EL, UMT, LMT and R).

As previously described in the literature [10], variations in tactical instruction could have affected the physical demands placed on soccer players in and out of ball possession. Distance in possession >24 km·h^−1^ was the only variable where the most successful teams obtained the highest values. Considering that sprints are the most important action in decisive offensive situations in soccer [18], the higher values achieved by CL could provide them an advantage in creating a greater number of these situations; thus having more opportunities to win matches, and by consequence, finishing higher in the league table. Although it has not been studied in this work, the different percentages of ball possession in the Spanish league (successful >52.8% vs. unsuccessful <48.9%) also could explain this outcome. Successful teams spend more time with the ball, allowing more opportunities to accumulate more meters in this variable. The conclusions of previous literature vary depending on the league studied. In the Italian Serie A, the best teams cover the greatest total distance and total distance above 14 and 19 km·h^−1^ with ball possession [15]; whilst in the English premier league, the worst and middle ranked teams covered a greater distance above 19.8 km·h^−1^ without the ball [12]. 

Our data did not discriminate between the physical performance of successful and unsuccessful teams in La Liga, casting doubt on the idea that worse teams have greater physical outputs than the best teams. These values of match activity can be understood to be important for a better ranking at the end of the League season, without forgetting that overall technical and tactical effectiveness probably have a greater impact on results and teams’ final league rankings [3]. 

This study presents some limitations. Firstly, it has only researched some physical data without including technical-tactical information or parameters gained by GPS micro-technology that allow us to assess performance in a more holistic manner. For this reason, we feel it would be interesting to replicate this study including technical-tactical data and accelerations, decelerations, player load, etc.

Likewise, another limitation present in this work is that data with and without possession were studied without taking into account the number or the effective time of possession in each group. Future studies should perform this analysis to verify how these data are modified.

Another important limitation is that the presented data show mean responses of a group of teams without explaining individual responses of the players in each team, so future literature should identify individual responses of each position in the chosen groups.

In summary, our results show that there were differences in physical performance in competitions between successful and unsuccessful teams in a highly competitive league season. These differences occur even among successful teams when they are classified in terms of final ranking (UEFA Champions League, UEFA Europa League and Upper Mid-Table). Equally important, is that our data indicates that unsuccessful teams do not always achieve a higher physical performance than successful teams, as has been established previously in the literature.

The present study shows that when teams are classified in terms of their final ranking, there are no differences in running demands between successful and unsuccessful teams. With this data in mind, it can be said that:

- Having a knowledge of the physical demands of the game allows coaches to prepare specific training that allows the players to cope with this demand. The present data helps them to obtain some guidelines on the performance of professional teams.
- Technical staff should ensure that their players can achieve the values necessary to achieve optimal performance in their league.
- Increasing the physical demands of the players, alongside their technical and tactical abilities, is a potentially suitable route to increasing team performance. Thus, training in a holistic manner where players improve their fitness with the ball through modified games, could be a recommended modality to achieve success in soccer.

## Figures and Tables

**Table 1:** Time-motion of the Spanish League teams in the season 2013–2014.

	Classifications of the Teams				
*Variable*	CL	EL	UMT	LMT	R
Total distance	10,137 ± 1407 ^U, MH, R^	10,509 ± 983	10,643 ± 980	10,323 ± 100 ^MH^	10,442 ± 971
Relative distance	108 ± 16 ^U, MH, R^	112 ± 11	113 ± 11	110 ± 11 ^MH^	111 ± 11
Distance >14 km·h^−1^	2411 ± 745 ^MH^	2505 ± 637	2643 ± 669	2415 ± 622 ^MH^	2478 ± 622
Distance 14–21 km·h^−1^	1928 ± 624 ^U, MH^	2026 ± 540	2154 ± 577	1970 ± 529 ^MH^	2013 ± 543 ^MH^
Distance <14 km·h^−1^	7726 ± 898 *	8005 ± 565	8000 ± 551	7908 ± 561	7958 ± 569
Distance >21 km·h^−1^	483 ± 229	478 ± 205	489 ± 207	445 ± 187	464 ± 193
Distance >24 km·h^−1^	238 ± 146	232 ± 137	232 ± 133	211 ± 123	223 ± 125
Distance in possession	3674 ± 890	3704 ± 792	3732 ± 837	3620 ± 813	3663 ± 776
Out Possession >14 km·h^−1^	1080 ± 509 ^MH^	1109 ± 493	1172 ± 475	1078 ± 491 ^MH^	1101 ± 478
Out Possession >21 km·h^−1^	225 ± 153	221 ± 150	231 ± 147	206 ± 137	220 ± 142
Out Possession >24 km·h^−1^	111 ± 98	108 ± 91	111 ± 88	98 ± 81	107 ± 85
Distance without possession	3919 ± 963 ^U, MH^	4134 ± 861	4132 ± 839	3870 ± 843 ^U, MH^	4048 ± 856
In Possession >14 km·h^−1^	1174 ± 537 ^U, MH^	1224 ± 498	1297 ± 508	1162 ± 513 ^U, MH^	1207 ± 503
In Possession >21 km·h^−1^	250 ± 170	247 ± 159	249 ± 153	229 ± 148	235 ± 146
In Possession >24 km·h^−1^	123 ± 105	121 ± 101	119 ± 93	110 ± 90 ^C^	113 ± 89

Note: Data represent means and standard deviations. CL = UEFA Champions League; EL = UEFA Europa League; UMT = Upper Middle Table; LMT = Lower Middle Table; R = Relegation; * = Substantial differences with all other teams; C = Substantial differences with Champions League Teams; U = Substantial differences with UEFA Europa League Teams; MH = Substantial differences with Upper Middle Table; R = Substantial differences with relegation teams.

**Table 2:** Effect size and *p* valor.

Comparison between Groups																				
	CL vs. EL		CL vs. UMT		CL vs. LMT		CL vs. R		EL vs. UMT		EL vs. LMT		EL vs. R		UMT vs. LMT		UMT vs. R		LMT vs. R	
Variable	ES	*p*	ES	*p*	ES	*p*	ES	*p*	ES	*p*	ES	*p*	ES	*p*	ES	*p*	ES	*p*	ES	*p*
Total Distance	0.3	0.00	0.4	0.00	0.0	0.00	0.3	0.00	0.1	0.00	−0.2	0.00	−0.1	0.25	−0.4	0.00	−0.3	0.00	0.1	0.01
Relative distance	0.3	0.00	0.4	0.00	0.2	0.00	0.2	0.00	0.1	0.00	−0.3	0.00	−0.1	0.07	−0.3	0.00	−0.2	0.00	0.1	0.02
In Possession	0.3	0.00	0.3	0.00	0.0	0.93	0.2	0.00	0.0	0.88	−0.3	0.00	−0.1	0.06	−0.3	0.00	−0.1	0.02	0.2	0.00
Without Possession	0.1	0.08	0.1	0.02	0.0	0.59	0.0	0.39	0.0	0.73	−0.1	0.01	−0.1	0.34	−0.1	0.00	−0.1	0.16	0.0	0.12
Distance >14 km·h^−1^	0.2	0.00	0.3	0.00	0.1	0.00	0.2	0.00	0.2	0.00	−0.1	0.09	0.0	0.75	0.2	0.00	0.0	0.75	0.1	0.01
Distance 14–21 km·h^−1^	0.2	0.00	0.4	0.00	0.2	0.00	0.2	0.00	0.2	0.00	−0.1	0.03	0.0	0.82	−0.3	0.00	−0.2	0.00	0.1	0.04
Distance <14 km km·h^−1^	0.4	0.00	0.4	0.00	0.3	0.00	0.3	0.00	0.0	0.92	−0.2	0.00	−0.1	0.13	−0.2	0.00	−0.1	0.10	0.1	0.05
Distance >21 km·h^−1^	0.0	0.40	0.1	0.03	0.1	0.15	0.0	0.63	0.1	0.21	−0.1	0.01	0.0	0.66	−0.2	0.00	−0.1	0.06	0.1	0.02
Distance >24 km·h^−1^	0.0	0.37	0.0	0.37	−0.2	0.00	−0.1	0.10	0.0	0.90	−0.1	0.01	0.0	0.50	−0.1	0.00	0.0	0.38	0.1	0.09
Out Possession >14 km·h^−1^	0.1	0.00	0.3	0.00	0.1	0.08	0.1	0.00	0.1	0.00	−0.1	0.11	0.0	0.84	−0.3	0.00	−0.2	0.01	0.1	0.17
Out Possession >21 km·h^−1^	0.0	0.84	0.1	0.18	−0.1	0.25	0.0	0.74	0.1	0.31	−0.1	0.21	0.0	0.90	−0.1	0.00	0.0	0.37	0.1	0.16
Out Possession >24 km·h^−1^	0.0	0.99	0.1	0.16	−0.1	0.10	0.0	0.83	0.1	0.22	−0.1	0.15	0.0	0.85	0.1	0.24	0.0	0.85	0.1	0.11
In Possession >14 km·h^−1^	0.2	0.00	0.3	0.00	0.1	0.21	0.1	0.00	0.1	0.02	−0.3	0.00	0.0	0.38	−0.3	0.00	−0.2	0.00	0.1	0.03
In Possession >21 km·h^−1^	0.0	0.74	0.0	0.91	−0.1	0.03	−0.1	0.16	−0.1	0.80	−0.1	0.02	−0.1	0.10	−0.1	0.01	−0.1	0.12	0.0	0.66
In Possession >24 km·h^−1^	0.0	0.64	0.1	0.20	−0.1	0.00	−0.1	0.35	0.0	0.48	−0.1	0.01	0.0	0.66	−0.1	0.04	0.0	0.82	0.1	0.04

Note: ES = Effect size; p = p valor. CL = UEFA Champions League; EL = UEFA Europa League; UMT = Upper Middle Table; LMT = Lower Middle Table; R = Relegation.
